# Effect of Inlet Flow Strategies on the Dynamics of Pulsed Fluidized Bed of Nanopowder

**DOI:** 10.3390/nano13020304

**Published:** 2023-01-11

**Authors:** Syed Sadiq Ali, Agus Arsad, Kenneth L. Roberts, Mohammad Asif

**Affiliations:** 1 School of Chemical and Energy Engineering, Faculty of Engineering, Universiti Teknologi Malaysia, Johor Bahru 81310, Johor, Malaysia; 2 UTM-MPRC Institute for Oil and Gas, School of Chemical and Energy Engineering, Faculty of Engineering, Universiti Teknologi Malaysia, Johor Bahru 81310, Johor, Malaysia; 3 SmartState Center for Strategic Approaches to the Generation of Electricity (SAGE), College of Engineering and Computing, University of South Carolina, Columbia, SC 29208, USA; 4 Department of Chemical Engineering, King Saud University, P.O. Box 800, Riyadh 11421, Saudi Arabia

**Keywords:** fluidization, pulsation, frequency, flow strategy, flow spike, disturbances

## Abstract

The use of fluidization assistance can greatly enhance the fluidization hydrodynamics of powders that exhibit poor fluidization behavior. Compared to other assistance techniques, pulsed flow assistance is a promising technique for improving conventional fluidization because of its energy efficiency and ease of process implementation. However, the inlet flow configuration of pulsed flow can significantly affect the bed hydrodynamics. In this study, the conventional single drainage (SD) flow strategy was modified to purge the primary flow during the non-flow period of the pulse to eliminate pressure buildup in the inlet flow line while providing a second drainage path to the residual gas. The bed dynamics for both cases, namely, single drainage (SD) and modified double drainage (MDD), were carefully monitored by recording the overall and local pressure drop transients in different bed regions at two widely different pulsation frequencies of 0.05 and 0.25 Hz. The MDD strategy led to substantially faster bed dynamics and greater frictional pressure drop in lower bed regions with significantly mitigated segregation behavior. The spectral analysis of the local and global pressure transient data in the frequency domain revealed a pronounced difference between the two flow strategies. The application of the MDD inlet flow strategy eliminated the disturbances from the pulsed fluidized bed irrespective of the pulsation frequency.

## 1. Introduction

Fluidized bed technology holds great promise for improving the process efficiency in the petrochemical and chemical industries. Its key strength lies in efficient solid dispersion that ensures effective utilization of the solids’ surface area and significantly enhances the surface-based rate processes. In the fluidized bed mode of contact, lower pressure drop, greater interfacial contact, efficient gas–solid mixing, and higher heat and mass transfer rates provide a substantial advantage over fixed bed or packed bed processes [1,2,3,4,5].

Particle properties have a strong bearing on fluidization behavior, as pointed out by Geldart, who classified the powders into different groups based on physical properties [6]. The fluidization of fine and ultrafine particles of group C classification is difficult because of their cohesiveness caused by strong inter-particle forces (IPF) [7,8], which often result in poor interphase phase mixing and severe bed non-homogeneities [9,10,11,12]. In particular, ultrafine powders display agglomerate bubbling fluidization (ABF) due to the formation of large multi-level agglomerates, leading to low bed expansion and high minimum fluidization velocity. As a result, the high surface area characteristics of ultrafine particles become severely compromised. Moreover, size-based segregation often occurs along the height of the bed during ABF [13,14,15,16,17]. The size of hydrophobic silica agglomerates in the lower bed region was 5–10 times larger than the ones in the upper layer [18]. Zhao et al. [19] reported more severe size segregation, where an order of magnitude agglomerate size difference between the upper and lower layers occurred, such that agglomerates as large as 2000 µm were found in the lower region. Although higher gas flow tends to mitigate the effect of IPFs, other problems, such as elutriation and entrainment, occur at high velocities. Therefore, various assistance strategies have been suggested in the literature to provide additional energy to overcome cohesive IPFs and improve the hydrodynamics of the fluidized bed. One such strategy is vibration assistance, which can be employed either internally or externally. External vibrations involve oscillating, shaking, or vibrating the complete test section by using a vibrator or an electric motor [19,20]. Despite their proven capability, the implementation of external vibration-based fluidization assistance techniques, whether at a laboratory scale or large-scale units, is challenging and expensive. On the other hand, internal vibrations directly transfer energy to the solid phase in the bed through techniques such as acoustic perturbation and high-shear mixer [21,22,23,24,25,26,27]. Similar to external vibrations, these techniques require additional equipment, leading to higher costs. In some cases, the premixing of the resident solid phase with inert or magnetic particles has been suggested to alter the interparticle force equilibrium of ultrafine particles in the bed [15,28,29,30,31]. The compatibility and post-processing issues, however, limit the application of particle premixing.

An important prerequisite for large-scale applications of any assisted technique is its amenability to scale up and easy implementation without being energy intensive and any major process modification requirement. One such technique is the pulsation of inlet fluid flow to the fluidized bed [32,33,34]. The flow pulsation shortened the drying time and improved the bed homogeneity in drying porous pharmaceutical granules [35]. The constant and falling drying rates of the fluidized biomass particles were enhanced by using optimal pulsation frequency [36]. The flow pulsation also promoted the density-based segregation of coal particles [37,38]. During the fluidization of ultrafine nanoparticles using the square wave pulsation strategy, the channeling and plug formation were suppressed, leading to improved bed homogeneity [16,33,39,40,41]. Moreover, the minimum fluidization velocity significantly decreased, indicating the deagglomeration of large-sized nanoagglomerates [16,39,40]. Besides promoting uniform bed expansion, pulsed flow also helped decrease the bubble velocity and size [40].

The efficacy of pulsed flow strongly depends on inlet flow and deaeration configurations. The effect of the deaeration strategy has been extensively studied in the context of bed collapse [42,43,44]. Once the inlet flow is stopped, the deaeration of the residual air critically affects the bed collapse process, which is clearly reflected in the evolution of pressure transient profiles in different regions of the bed [43,44]. The ratio of the distributor to bed pressure drop showed a pronounced effect on the collapse process. As this ratio was increased from 0.005 to 0.03, the difference between the two deaeration strategies was substantially mitigated [43]. When the residual air escapes only from the top of the bed, known as single drainage (SD) deaeration, the collapse process is slow. Providing the residual air dual pathways (i.e., from the top as well as from the bottom of the bed through the plenum), also called dual drainage (DD) deaeration, leads to faster bed transients [42]. Of equal importance is the design of the inlet flow configuration because the line pressure inevitably builds up during the no-flow phase of the pulse when the bed collapses. Therefore, once the valve opens to allow the inlet flow, the line pressure leads to a flow spike. This phenomenon leads to intense size segregation of nanoagglomerates along the height of the fluidized bed, thereby affecting the collapse dynamics monitored in different regions of the bed. To suppress the initial flow spike by eliminating the line pressure buildup, Ali et al. suggested to vent the inlet flow to the atmosphere during the no-flow phase of the pulse while allowing dual deaeration routes to residual gas. This modified dual drainage (MDD) strategy significantly suppressed the size segregation of agglomerates and improved the bed homogeneity [18].

The foregoing discussion, although mainly in the context of bed collapse, is of great importance for pulsed fluidized beds. The flow spike resulting from the pressure buildup during the no-flow phase of the square pulse could severely compromise the efficacy of pulsation assistance. This phenomenon could be complicated by pulsation frequency. At a shorter pulsation frequency, the line pressure builds up because of the longer duration of the no-flow phase of the pulse. Therefore, a careful investigation has been undertaken using the MDD strategy for the fluidized bed pulsed with two widely different square wave frequencies, namely, 0.05 and 0.25 Hz. The local and global bed dynamics for both frequencies were monitored using highly sensitive pressure transducers with a response time of 1 ms. The results were compared with SD pulsed bed to obtain a greater understanding of the pulsed fluidized bed. The present study substantially extends the scope of the conventional bed collapse to examine how the regular intermittency of bed collapse affects the evolution of local and global bed transients in the presence of two different deaeration strategies. Modifying the inlet configuration to suppress the initial peak adds another dimension to the problem, that is, how the occurrence of a short-term event affects the subsequent development of fluidization hydrodynamics.

## 2. Experimental

**Figure 1:**
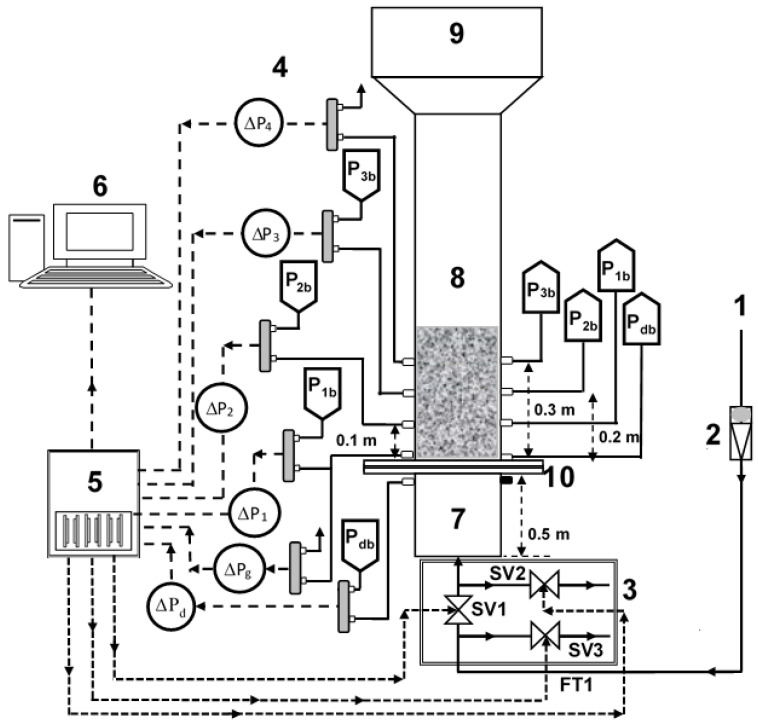
Experimental set-up; (1) Compressed air; (2) Flowmeter; (3) 2-way solenoid valves (for SD and MDD flow strategy); (4) Pressure transducers; (5) Data acquisition system; (6) Computer; (7) Calming section; (8) Test section; (9) Disengagement section; (10) Distributor.

The experimental setup consisted of a test section that was a 1.6 m long transparent perplex column with a 0.07 m internal diameter (Figure 1). A calming section with a length of 0.3 m was attached beneath the test section to eliminate the effects of the entry of the fluidizing air. A distributor with 0.025 fractional open area and 2 mm perforations on a circular pitch was used to ensure a uniform distribution of the fluidizing gas across the cross-sectional area of the test section. The perforations of the distributor were covered by a fine nylon mesh filter of 20 μm to prevent the falling of particles through the distributor. A disengagement section with a length of 0.5 m and a diameter of 0.14 m was attached above the test section to suppress particle entrainment with the exiting gas. The test section was washed with an anti-static fluid prior to the experiments.

The overall and local pressure drop transients in four different regions of the bed were measured by positioning the pressure taps along the bed height (Table 1). Five highly sensitive bidirectional differential-pressure transducers (Omega PX163-005BD5V; 1 ms response time; range: ±2.5″ H_2_O) recorded the pressure transients at a rate of 100 Hz by using data acquisition system (DAQ) and Labview software. The lower and upper ports of pressure transducers were located diametrically opposite sides of the column to ensure reliable monitoring of cross-sectionally averaged local bed dynamics in different bed regions (Figure 1).

The primary dimension of hydrophilic nanosilica (Aerosil 200) reported by the manufacturer (Evonik, GmBH) was 12 nm with a density of 2200 kg/m^3^ [45]. However, the dry particle size analysis (Malvern Panalytical Mastersizer 2000) yielded an average size of 12.5 μm due to the multi-level agglomeration of particles under the effect of IPFs [1,24]. This behavior was clearly evident in the morphological characterization of the nanopowder sample by SEM [13,16]. The specific surface area of the powder was 0.62 m^2^/g, which was several orders of magnitude smaller than the reported value of 200 ± 25 m^2^/g [45].

Three different two-way solenoid valves (Model: Omega SV 3310) were used (Figure 1). These valves were controlled using digital IO signals from the DAQ to provide two different inlet flow strategies during pulsation [18].

### 2.1. Single Drainage (SD) Configuration

In this configuration, only the primary inlet valve, marked as SV1 in Figure 1, was employed. When energized, SV1 allowed the inlet airflow and stopped it when de-energized. The other two valves, namely, SV2 and SV3, remained closed throughout the experiment. This strategy provided only one passage, that is, the top of the bed, for the escape of the trapped residual air during the collapse process. Since the square wave flow pulsations were implemented in our experiments, the closing of the valve during the no-flow phase of the square wave led to the buildup of the line pressure across SV1. This pressure buildup led to an initial airflow spike when the valve opened to allow the inlet flow to the test section.

### 2.2. Modified Dual Drainage (MDD) Configuration

In this configuration, SV1 was kept open during inlet flow to the test section, while the two other valves (SV2 and SV3) remained closed. However, when SV1 closed, cutting off the airflow, SV2 and SV3 were energized to remain fully open. SV2 provided an alternate passage for the residual air to escape through the plenum, while SV3 vented the primary airflow to the atmosphere, thereby preventing the buildup of the pressure drop. This strategy completely eliminated the initial airflow spike when SV1 opened to allow the inlet flow to the test section.

The opening and closing frequency of the solenoid valves was controlled using a digital IO signal from DAQ and Labview. High-pressure air under ambient conditions was used as the fluidizing gas. Gilmont flowmeters were used to set the initial airflow in the column. The particle bed was allowed to achieve a steady state before the start of the pulsation experiments using two different frequencies, namely 0.05 and 0.25 Hz. Whereas 0.05 Hz pulsations with a time period of 20 s allowed the complete collapse of the bed between two successive pulsations [13], 0.25 Hz pulsations with a much shorter time period of 4 s allowed only partial bed collapse before the occurrence of another pulsation event [1]. Local and global bed dynamics were monitored for four identical pulses of 0.05 Hz and six pulses of 0.25 Hz pulsation.

## 3. Results and Discussion

### Evolution of Local Pressure Drop Transients

**Figure 2:**
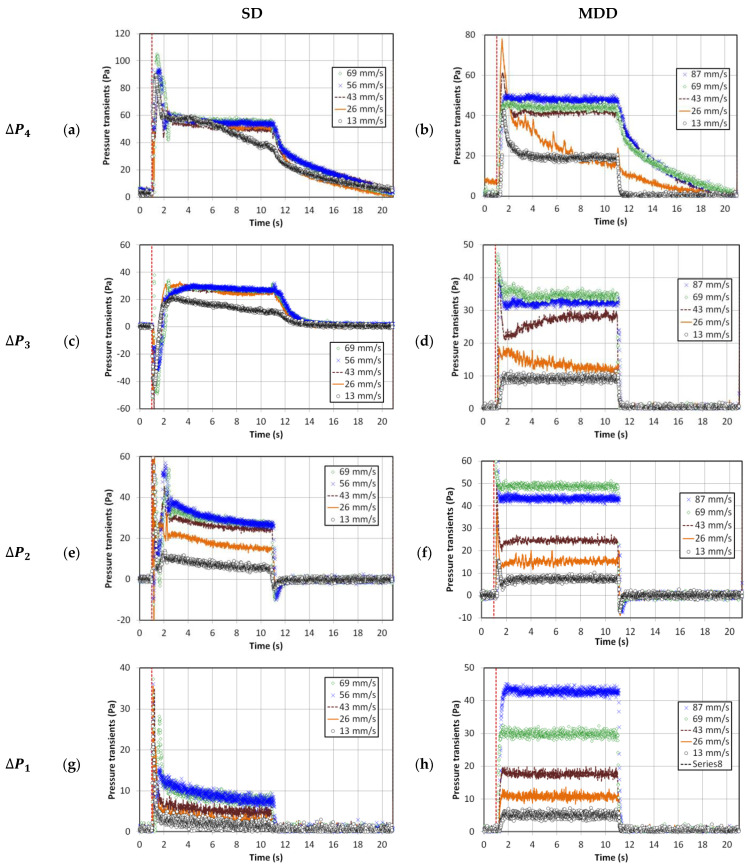
Local pressure drop transients with pulsation frequency 0.05 Hz; (**a**) Upper region (SD); (**b**) Upper region (MDD); (**c**) Upper middle region (SD); (**d**) Upper middle region (MDD); (**e**) Lower middle region (SD); (**f**) Lower middle region (MDD); (**g**) Lower region (SD); (**h**) Lower region (MDD).

Figure 2 shows the local bed dynamics of the 0.05 Hz pulsed fluidized bed under different inlet and deaeration strategies (i.e., SD and MDD). The broken red vertical line indicates the start of the flow pulse, while the collapse process theoretically initiates as soon as the inlet flow is stopped during the no-flow phase of the pulse. At 0.05 Hz, the complete pulse cycle lasted for 20 s that comprised 10 s of inlet flow at a fixed velocity, followed by 10 s of complete flow interruption. A wide spectrum of velocities within 13–87 mm/s were considered in our experiments. The peaks at the onset of the flow pulse, often pronounced for SD configuration, were due to the pressure buildup across the solenoid valve, resulting in the initial flow spike. This phenomenon can promote size-based segregation along the height of the bed [18]. By contrast, the venting of the primary flow in the MDD strategy did not allow the line pressure buildup, thereby suppressing the initial flow spike.

The effect of velocity variation on the SD fluidized bed was not always notable, except in the lower middle region that is represented by $\Delta P_{2}$ in Figure 2e. Since the bed was not fluidized at 13 mm/s, its dynamics inevitably differed from others. Unlike SD, the effect of velocity on the local dynamics of the MDD fluidized bed was significantly pronounced with substantially faster dynamics. Once the pulsed flow began, the pressure drop almost attained a steady state value within a span of 1 s. The collapse process also finished in one second, with the pressure drop attaining a zero value. However, some exceptions were seen for the upper region ($\Delta P_{4}$) and the upper middle region ($\Delta P_{3}$) of the bed. The faster dynamics of the MDD strategy could be due to the availability of second escape routes for the residual air through the plenum in addition to the top of the bed. Being closer to the lower drainage pathway, the difference between the two strategies in behavior was inevitably more pronounced in the lower region (Figure 2g,h). The evolution of pressure transients and their dependence on velocity in Figure 2h clearly highlight the improved bed hydrodynamics obtained using the MDD strategy. However insignificant, the initial pressure drop spike in the MDD pulsed bed could have resulted from the time lag in the closing of the vent valve and the opening of the flow valve.

In the lower bed region (Figure 2g), the accumulation of rigid and large agglomerates due to segregation resulted in a lower pressure drop than that in the regions above, that is, $\Delta P_{2}$ to $\Delta P_{4}$. However, in Figure 2h, the pressure drop was comparable with that in the middle region with a strong dependence on the velocity, which indicated the presence of smaller agglomerates in the lower region due to feeble segregation tendencies.

**Figure 3:**
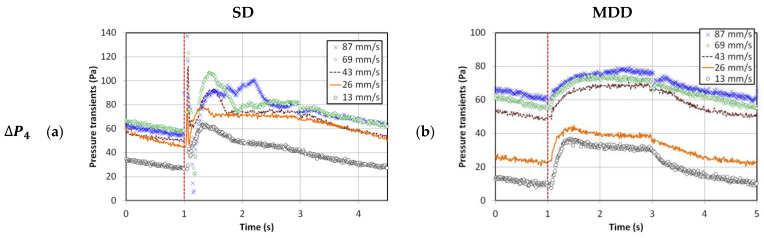

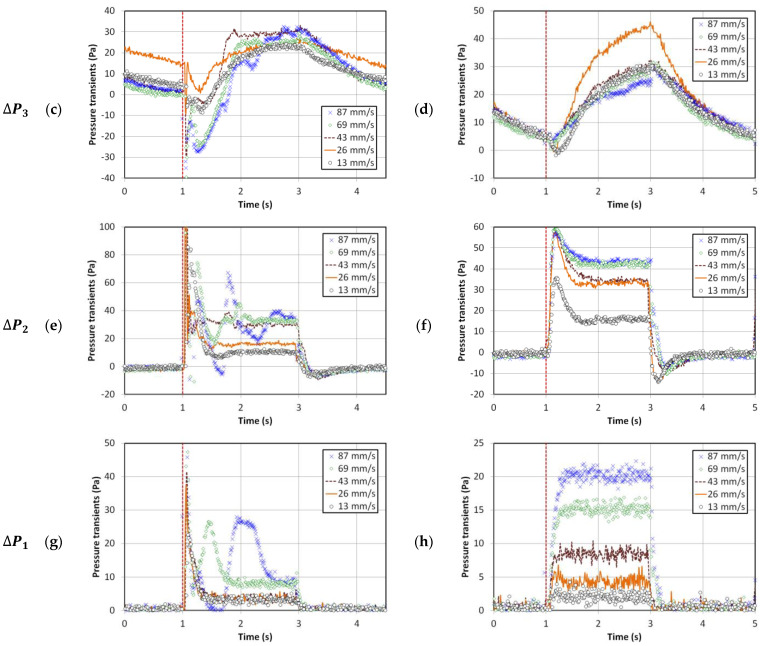
Local pressure drop transients with pulsation frequency 0.25 Hz; (**a**) Upper region (SD); (**b**) Upper region (MDD); (**c**) Upper middle region (SD); (**d**) Upper middle region (MDD); (**e**) Lower middle region (SD); (**f**) Lower middle region (MDD); (**g**) Lower region (SD); (**h**) Lower region (MDD).

The behavior of the higher frequency pulsed fluidized bed is shown in Figure 3. The inlet flow occurred for 2 s only, followed by 2 s of complete interruption. Therefore, neither the expansion nor the collapse process could reach a steady condition in most cases, irrespective of whether SD or MDD strategy was implemented. The evolution of MDD pressure transients were rather predictable, whereas a great deal of disturbances was evident for the SD transients. These disturbances were strongly affected by the change in velocity. In the case of MDD, $\Delta P_{1}$ and $\Delta P_{2}$ showed faster transients than $\Delta P_{3}$ and $\Delta P_{4}$ owing to the availability of the lower deaeration route through the plenum. Away from the distributor, the transients were slower, especially at higher velocities, because of the presence of a greater amount of residual gas. On the other hand, the complex, unpredictable pressure drop transients in the SD pulsed fluidized bed developed due to the interaction between the solid particles, whether rising or falling and the upward rising gas flow through the bed. The difference between the local bed dynamics in the two cases of different frequencies was also observed in the initial pressure drop spike, which appeared to be significantly mitigated for the higher frequency pulsed flow owing to a lower line pressure buildup due to the shorter duration of the no-flow phase of the pulsed flow.

The pressure drop ranges were of comparable magnitude in the lower region $\left(\Delta P_{1}\right)$ for both strategies at higher frequency pulsation in Figure 3g,h. Moreover, $\Delta P_{1}<\Delta P_{2}$, suggesting similar segregation behavior for both flow strategies. At higher frequencies, although the pulsed bed with SD configuration showed a smaller initial flow spike, their hydrodynamics were more susceptible to intense disturbances.

**Figure 4:**
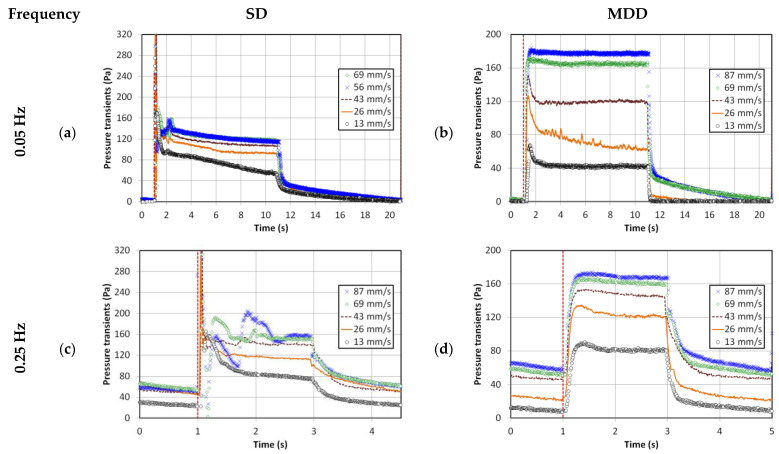
Global pressure drop transients for different pulsation frequencies; (**a**) 0.05 Hz frequency SD; (**b**) 0.05 Hz frequency MDD; (**c**) 0.25 Hz frequency SD; (**d**) 0.25 Hz frequency MDD.

The global transients are shown in Figure 4 for both cases of 0.05 and 0.25 Hz pulsed fluidized bed. The trends in Figure 2 and Figure 3 were witnessed again. The smooth pressure transients for MDD, irrespective of the pulsation frequency, are a clear reflection of the improved hydrodynamics. At lower frequencies, another aspect of the bed hydrodynamics that was apparently not evident before occurred. Higher pressure drop values were noticed at higher velocities, such as 69 and 87 mm/s, indicating better contact between the solid and fluid phases causing greater frictional losses.

**Figure 5:**
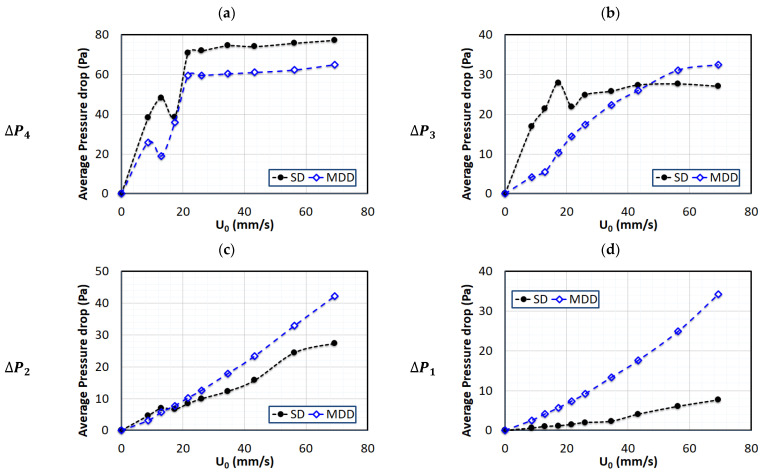
Variations in local pressure drop with air superficial velocity for defluidization runs of 0.05 Hz pulsed fluidized bed; (**a**) Upper region ($\Delta P_{4}$); (**b**) Upper middle region ($\Delta P_{3}$); (**c**) Lower region ($\Delta P_{2}$); (**d**) Lower region ($\Delta P_{1}$).

The bed hydrodynamics were further investigated by computing the mean value of the pressure drop from the pressure transients. The latter portion of the flow pulse, immediately before the flow cutoff, was used to evaluate the mean. The case of 0.05 Hz pulsed fluidized bed is considered in Figure 5. Only the defluidization part of the experimental run was compared for SD and MDD, owing to its repeatability. The difference was significantly pronounced in the lower region of the bed ($\Delta P_{1}$), which was monitored in the bed region from 5–100 mm above the distributor (Figure 5d). At higher flowrates, the difference between the two pressure drop values reached several folds with a steeper rise for the MDD, indicating the presence of smaller agglomerates. A similar difference, albeit less pronounced, was again observed for $\Delta P_{2}$ (Figure 5c). A smoother pressure drop profile was seen with the MDD in the upper middle region (Figure 5b). The upper region, represented by $\Delta P_{4}$, fully fluidized at approximately 20 mm/s, with a higher pressure drop for SD. Given that the total weight of solid particles in both cases were the same, the total pressure drops across the bed for the fully fluidized bed in both cases should be comparable. The lower pressure drop values in the lower region obtained with SD were therefore compensated in the upper region of the bed. Owing to the size segregation of nanoagglomerates, the bed showed partial fluidization. The upper region with smaller agglomerates fluidized at 20 mm/s, whereas the lower region with large agglomerates remained un-fluidized even at higher gas velocities.

**Figure 6:**
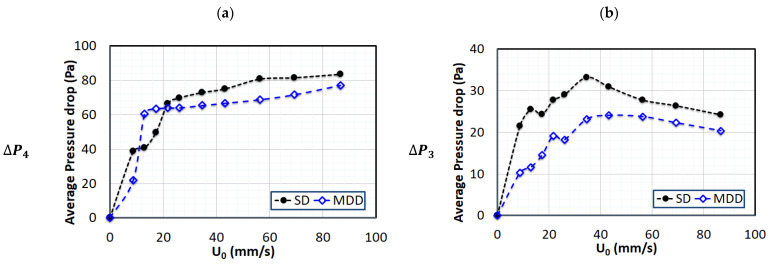

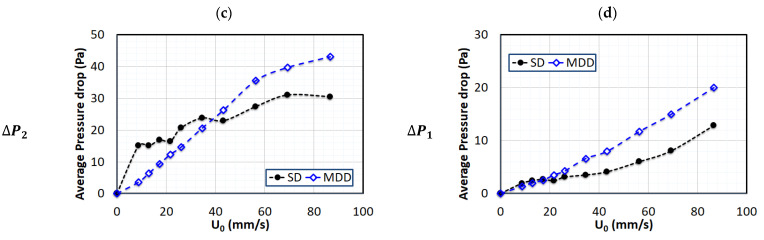
Variations in local pressure drop with air superficial velocity for defluidization runs of 0.25 Hz pulsed fluidized bed; (**a**) Upper region ($\Delta P_{4}$); (**b**) Upper middle region ($\Delta P_{3}$); (**c**) Lower region ($\Delta P_{2}$); (**d**) Lower region ($\Delta P_{1}$).

Similar behavior was observed for the case of 0.25 Hz pulsed bed in Figure 6. A clear difference between SD and MDD was detected in the lower region, where a higher pressure drop was obtained with MDD, and the difference between the two strategies was more pronounced at higher velocities. However, the difference was not as prominent as it was in the lower frequency case. The trend was reversed for the upper middle region ($\Delta P_{3}$) and the upper region ($\Delta P_{4}$) due to the material balance consideration, as explained in the preceding paragraph. The MDD bed hydrodynamics appeared to be more sensitive to the frequency change. The upper middle region showed a decrease in the pressure drop at higher velocities due to the bed expansion that caused the migration of solids to the upper region, where this phenomenon was reflected in the increase in the pressure drop.

**Figure 7:**
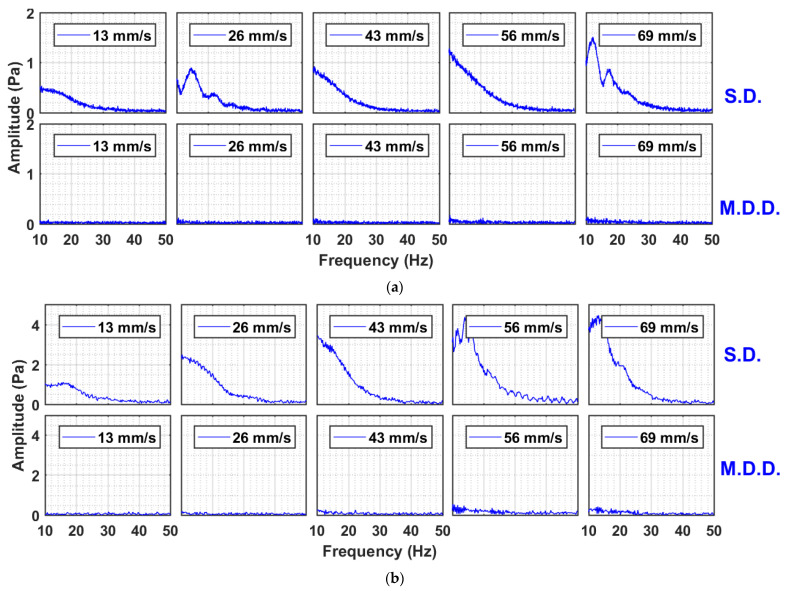
Amplitude spectral analysis of global pressure drop transients of SD and MDD pulsed fluidized bed at (**a**) 0.05 Hz; (**b**) 0.25 Hz.

A more revealing insight into the bed dynamics is shown in Figure 7, which presents the amplitude spectra of the signals in the frequency domain. The “fft” function of MATLAB was used for computing the spectra. The figure considers the global pressure drop signals that include the disturbances in the whole bed. The comparison for both cases of SD and MDD is also shown. The case of the 0.05 Hz pulsed bed is considered in Figure 7a for different velocities. The difference between the SD and MDD was significant. The amplitude spectra of the pressure drop transients of the SD within the 10–50 Hz range were dominated by the small amplitude events, which decreased in intensity as the frequency increased. This finding clearly indicated the disturbances occurring in the pulsed fluidized bed with SD configuration. The amplitude profile showed the existence of a wide spectrum of pressure fluctuations beginning from below 10 Hz and extending up to 30 Hz. It was caused by the solid particles falling under gravity and were obstructed by the upward flow of the residual air exiting from the top of the bed, thereby generating pressure drop fluctuations. This phenomenon was completely absent in the case of MDD. The case of 0.25 Hz pulsed bed is considered in Figure 7b. The behavior was similar to the one observed earlier for 0.05 Hz. The amplitude of fluctuations for SD at this frequency was higher than that obtained for the case of the lower frequency. This fact was already pointed out while discussing the real-time bed dynamics of SD configuration in Figure 3.

**Figure 8:**
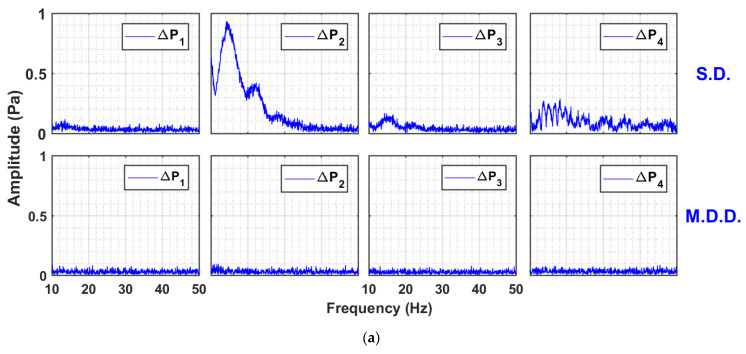

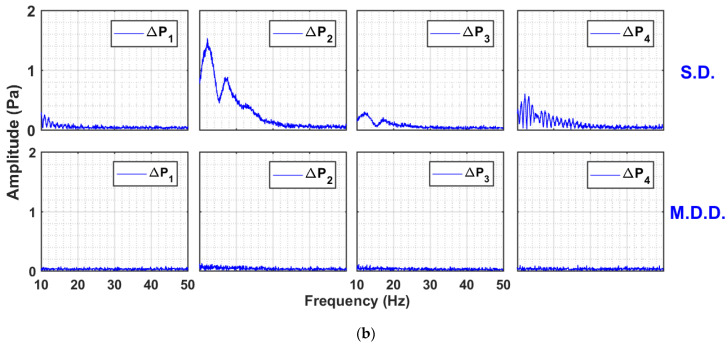
Amplitude spectral analysis of local pressure drop transients of 0.05 Hz pulsed fluidized bed for one complete pulse at (**a**) $U_{0}=26\;\mathrm{mm}/s$; (**b**) $U_{0}=69\;\mathrm{mm}/s$.

To obtain further insight into the local bed dynamics, the local amplitude spectra of pressure transients in different regions of the bed at 0.05 Hz flow pulsation are shown in Figure 8 for two different velocities (i.e., 26 and 69 mm/s). Interestingly, the difference between the local dynamics was notable. The case of lower velocity, that is, 26 mm/s, is considered in Figure 8a. The lower region represented by $\Delta P_{1}$ showed no fluctuations because large and hard agglomerates in the lower region were still stationary at 26 mm/s; therefore, no visible change occurred whether the flow was started or cutoff. The lower middle region pressure transients ($\Delta P_{2}$) were greatly affected as the falling particles achieved higher kinetic energy by traversing a greater distance in the bed in reaching this region when obstructed by the upwards moving residual air, indicating a great deal of fluctuations. For the same reason, this effect was substantially mitigated in the upper middle region ($\Delta P_{3}$) because of the lower kinetic energy of falling solids. In the upper region, we observed fluctuations with a lot of distinct frequencies as the smaller particles interacted with the residual air exiting the bed as small bubbles. As the velocity was increased to 69 mm/s, the phenomenon described became more intense (Figure 8b).

**Figure 9:**
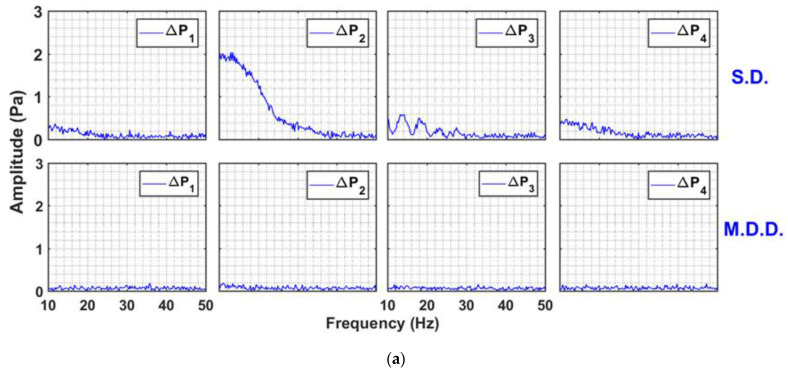

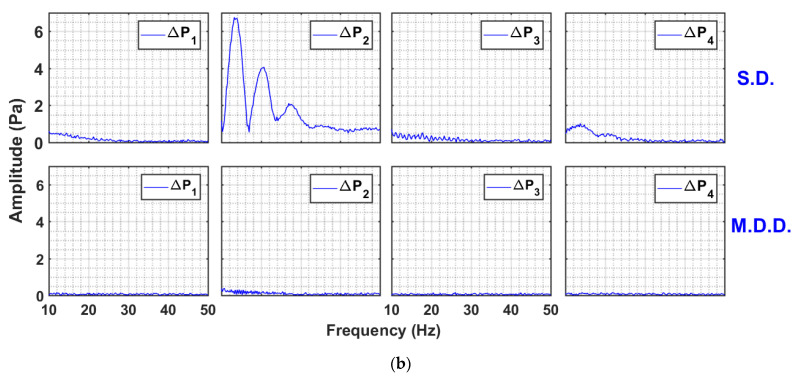
Amplitude spectral analysis of local pressure drop transients of 0.05 Hz pulsed fluidized bed for one complete pulse at (**a**) $U_{0}=26\;\mathrm{mm}/s$; (**b**) $U_{0}=69\;\mathrm{mm}/s$.

Similar observations were persistent at high-frequency pulsations (Figure 9a,b). The MDD configuration completely eliminated the disturbances throughout the bed, irrespective of gas velocity. For the SD configuration, the local bed hydrodynamics presented a completely different picture. The amplitude was the highest in the $\Delta P_{2}$ region, where the swarm of falling particles possessed the highest kinetic energy. In the region above, that is, upper middle, $\Delta P_{3}$ showed clear periodic events of small amplitude in the range of 10–20 Hz. Increasing the fluid velocity increased the amplitude by more than threefold due to the increased disturbances in the bed. The continuous spectra of $\Delta P_{2}$ at 26 mm/s changed into discrete high amplitude events occurring at 12 Hz followed by those at 20 Hz, probably due to the development of flow structures.

**Figure 10:**
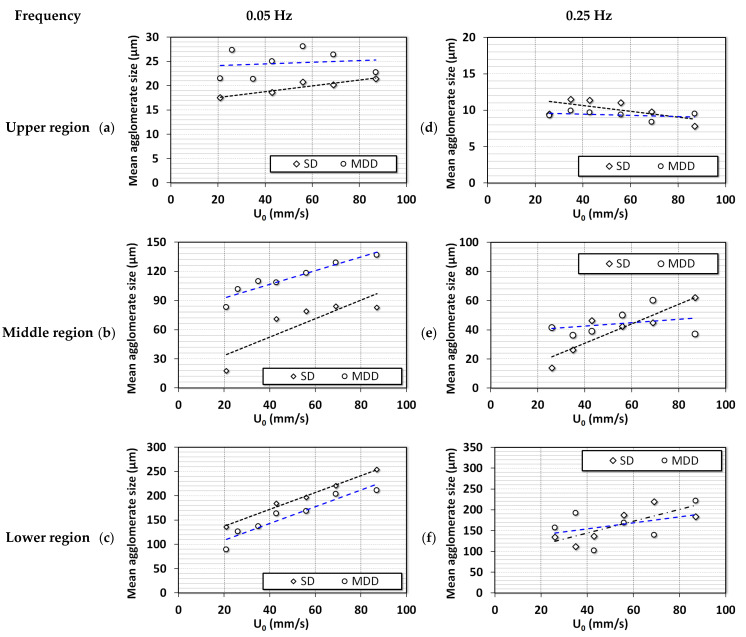
Changes in the average agglomerate diameter of ultrafine particles with the change in fluid velocity in different regions of the bed of nanosilica; (**a**) Upper region (0.05 Hz); (**b**) Middle region (0.05 Hz); (**c**) Lower region (0.05 Hz); (**d**) Upper region (0.25 Hz); (**e**) Middle region (0.25 Hz); (**f**) Lower region (0.25 Hz).

The average diameters from local dynamics were calculated during the collapse of the bed. The mathematical model based on mass balance proposed by Nie and Liu was used [46]. The diameters of the agglomerates are reported in Figure 10. For 0.05 Hz in Figure 10a–c, the segregation was clearly visible for SD configuration. The range of diameters for upper, middle, and lower regions were 15–22, 30–90, and 140–260 μm, respectively. The agglomerate size in the upper region was almost constant with airflow rate, while agglomerates in the middle and lower regions showed a consistent increase with airflow rate because finer particles moved to the upper region due to segregation. For the case of MDD configuration, the size ranges were 20–30, 90–140, and 100–200 μm in the upper, middle, and lower regions, respectively. The size difference between the agglomerates in the lower and middle regions was not as pronounced as those in the case of the SD configuration. The MDD configuration suppressed the segregation tendencies in the pulsed fluidized beds. At higher frequencies (Figure 10d–f), the size ranges were similar for both flow strategies with strong segregation behavior that was seen for the case of 0.05 Hz SD configuration. This means that the disturbances generated due to high-frequency pulsation developed a similar impact on the hydrodynamics as the initial airflow spike in the lower frequency. Moreover, the slope of the curve in Figure 10d decreased with increasing airflow, signifying the addition of finer particles in the region. Moreover, the slope of curves in Figure 10e,f was lower than that in Figure 10b,c. This finding could be due to the deagglomeration phenomenon, wherein the size of larger particles decreased, and finer particles moved to the upper region.

## 4. Conclusions

The inlet flow strategies greatly affected the hydrodynamics of pulsed fluidized beds. At a low pulsation frequency of 0.05 Hz, large size-based segregation was observed when the SD flow strategy was used. This was caused by the initial airflow spike resulting from pressure built up across the solenoid valve in the collapse process. This phenomenon was eliminated using the MDD airflow strategy, leading to a subdued segregation behavior. The region-wise pressure drop during defluidization displayed the difference in the degree of segregation, especially in the lower region due to the different flow strategies. However, at a higher pulsation frequency of 0.25 Hz, the flow spike was feeble due to the lower time period of pressure built up across the solenoid valve. The difference in stratification was less between both flow strategies when high-frequency pulsation was used. The particle diameter calculated from the local bed dynamics signified that the segregation was prominent for both flow strategies at high-frequency flow pulsation. The disturbances developed due to the frequent expansion and collapse of the bed promoted segregation and deagglomeration.

## Figures and Tables

**Table 1:** Pressure tap positions used to record region-wise pressure transients.

Pressure Drop	Bed Region	Pressure Tap Positions (from the Distributor)
** $\Delta P_{1}$ **	Lower	0.5–0.1 m
** $\Delta P_{2}$ **	Lower middle	0.1–0.2 m
** $\Delta P_{3}$ **	Upper middle	0.2–0.3 m
** $\Delta P_{4}$ **	Upper	0.3 m–open
** $\Delta P_{g}$ **	Overall	0.05 m–open
